# Elder abuse/mistreatment and associated covariates in India: results from the Longitudinal Aging Study in India wave 1, 2017-2018

**DOI:** 10.4178/epih.e2022017

**Published:** 2022-01-18

**Authors:** Thennavan Sathya, Yesuvadian Selvamani, Rangasamy Nagarajan

**Affiliations:** Department of Development Studies, International Institute for Population Sciences (IIPS), Mumbai. India

**Keywords:** Domestic violence, Depression, Multimorbidity, Functional limitation

## Abstract

**OBJECTIVES:**

Elder abuse has significant adverse consequences for the overall health and well-being of the elderly, including premature mortality. Using cross-sectional data, we assessed the prevalence of elder abuse in India, its variation across states, and associated factors.

**METHODS:**

Nationally representative data from the first wave of the Longitudinal Aging Study in India were analyzed. Bivariate and multivariate analyses were used to study the prevalence, state variations, and associated factors of elder abuse.

**RESULTS:**

Overall, 5.2% of elderly adults (≥60 years) had experienced abuse in the year prior to the survey and 3% had experienced abuse within their own household. Verbal abuse or disrespect was the most common form of abuse. Considerable variation was observed in the prevalence of elder abuse across states and union territories, with the highest prevalence observed in Bihar (11.6%) and Karnataka (10.1%). In regression analysis, education level emerged as a protective factor against elder abuse, particularly among women. Older adults who lived alone, had functional limitations, had multiple morbidities, and had been hospitalized in the past year were more likely to experience abuse. Older adults who experienced abuse were 2 times more likely to experience depressive symptoms.

**CONCLUSIONS:**

Cross-state variation in the prevalence of elder abuse and subgroup differences suggest that state-specific interventions and essential monitoring of older adults with functional limitations, chronic diseases, and recent hospitalization can further reduce the prevalence and consequences of elder abuse in India.

## INTRODUCTION

The World Health Organization (WHO) defines elder abuse or maltreatment as “a single, or repeated act, or lack of appropriate action, occurring within any relationship where there is an expectation of trust, which causes harm or distress to the older person” [1]. Elder abuse or ill-treatment is one of the key public health issues affecting a large proportion of the older global population. Globally, the overall prevalence of elder abuse is 15.7%, with significant differences across countries [2]. As a public health problem, elder abuse is associated with higher mortality [3-5], psychological distress, depression [6-8], hospitalization [9], and lower subjective well-being [10].

Existing studies have identified key individual and contextual characteristics contributing to elder abuse. Cultural factors such as age discrimination at the household and community level are closely associated with elder abuse [11,12]. At the individual level, family characteristics and socioeconomic status are significant predictors of elder abuse. Notably, education has been shown to be a protective factor for elder abuse [13,14]. Some studies observed a higher level of elder abuse among women [15,16], and age-associated factors such as cognitive impairment, dementia, and other chronic diseases or functional limitations were closely linked with abuse [17]. Functional independence is a key aspect of healthy aging. However, age is closely associated with functional limitations and a high disease burden, with subsequent dependency, healthcare utilization, and high hospital expenditures [18]. Studies have shown that age-related changes in health such as chronic disease, multimorbidity, and functional limitations are closely linked to elder abuse [17,19-21] Dependence on others to fulfill basic daily needs and high healthcare expenditures increase the vulnerability of elders. Factors such as frailty and dementia have also been linked with elder abuse [22,23].

Elder abuse is an important issue and is growing among the older population due to an increasing proportion of older adults in the population. Most studies on elder abuse have been conducted in high-income countries [24]; therefore, data on the factors associated with elder abuse in India is limited. A few studies have examined the role of education [13,14] and health on elder abuse or ill-treatment [19-21] in India. However, these studies were based on small samples or were region-specific. To our knowledge, no previous study has presented state-level variations in the prevalence of elder abuse for all states/union territories (UTs) in India. It is also necessary to understand the different factors related to elder abuse in India where variations in demographic and socioeconomic development among states are evident. Understanding the variations among states will help policy makers implement state-specific interventions.

India is experiencing rapid aging of its population, mainly due to improvements in health and reductions in fertility. As a result, the share of the older population is increasing [25] and an age-related rise in the prevalence of chronic diseases and functional disability is evident [26,27]. In addition, a majority of older adults in India are not educated and are poor [28,29], depending on family members for economic support [30]. Higher levels of widowhood among women are also evident in India, primarily because of the age gap between husbands and wives [31]. Previous studies have highlighted gender differences in health and cognition, reporting that women experienced a higher disability and disease burden than men and had lower performance scores in cognitive function testing [32,33]. However, the gender difference in elder abuse is less clear [14]. Considering these demographic-related and gender-related issues, understanding what contributes to elder abuse will be useful for establishing public policy in India. In addition, socio-cultural differences across states are notable and may play a significant role in determining caregiving options and impact the rates of elder abuse. However, no previous study has shown the influence of interstate variations on elder abuse in India by investigating health-related factors, including depressive symptoms and healthcare utilization. A large proportion of the older population is poorly educated and has been diagnosed with various health issues, functional limitations, and dementia. Understanding how this correlates with elder abuse and its effects will be useful in the context of an aging population in India. Therefore, this study assessed state variations and the correlates of elder abuse using recent data on older adults aged 60 years and above.

## MATERIALS AND METHODS

In this study, we used data collected from wave 1 of the Longitudinal Aging Study in India (LASI, 2017-2018), which represented all 35 states and UTs of India except for Sikkim. It is India’s first and largest nationally representative survey on middle-aged and older adults, and it provides detailed information on key measures such as the economic, social, and health status of older adults aged 45 years and above, including elder abuse and age discrimination. The LASI survey sample included 72,250 people. This study considered the data of adults aged 60 years and above since the abuse-related information was collected only on adults aged 60 and above. Therefore, the present study analyzed the data of 31,464 older adults aged 60 years and above. The LASI was undertaken in collaboration with the Harvard T. H. Chan School of Public Health and the University of Southern California, with major sponsorship of the Ministry of Health and Family Welfare, Government of India, and the United Nations Population Fund-India. More information about the survey and methodology is available in the 2020 LASI report [34]

### Measures

#### Outcome variable

Elder abuse was assessed with the survey question “Have you felt that you were ill-treated in the past year?” with a “yes” answer indicating abuse. Those who answered “yes” were then asked additional questions to determine the type of ill-treatment (physical abuse, verbal abuse/disrespect, economic exploitation, or emotional/psychological abuse and neglect) and the place of ill-treatment (within the home or outside the household).

#### Covariates

Our survey included items on selected socio-demographic characteristics such as age group, place of residence, gender, marital status, education, work status and household monthly per capita consumer expenditure (MPCE) quintile. Information on 6 activities of daily living (ADLs) was included (dressing, walking, bathing, eating, getting in or out of bed, and using the toilet) as correlates of abuse. We generated a single variable by combining all 6 measures and recoded the variables as no ADL limitations, 1 ADL limitation, 2 ADL limitations, and 3+ADL limitations. We also created a variable to measure multimorbidity by combining 9 self-reported chronic diseases (hypertension, diabetes, cancer, chronic lung disease, chronic heart disease, stroke, arthritis, any neurological or psychiatric problem, and high cholesterol) and generated a single variable coded as no disease, 1 disease, 2 diseases, or 3+ diseases.

#### Depressive symptoms

In this study, the Center for Epidemiological Studies Depression (CESD) 10-item scale was used to assess depressive symptoms. The scale includes 7 negative items and 3 positive items. Scoring was reversed for positive symptoms. The negative indicators included trouble concentrating, feeling depressed, low energy, fear of something, feeling lonely, bothered by things, and everything is an effort. The positive indicators included feeling happy, hopeful, and satisfied. The scores were summed (range, 0-10), and those who reported 4 or more symptoms were considered to have depressive symptoms. Previous studies have tested the validity of the CESD scale in the Indian setting [35].

### Statistical analysis

We conducted bivariate and multivariate analyses to understand the association of socio-demographic factors, chronic diseases, and functional health with elder abuse in India. Bivariate analysis was used to estimate the prevalence of elder abuse across states/UTs and socio-demographic and health variables. Multivariate logistic regression was used to assess the correlates of elder abuse. The association between elder abuse and depressive symptoms was also assessed using a multivariate logistic regression model.

### Ethics statement

This study used the secondary data collected by the International Institute for Population Sciences, Mumbai. The Indian Council of Medical Research approved the survey and a consent form was signed by all age-eligible respondents.

## RESULTS

In this study, more than 40% of the respondents were above 70 years and more than 50% of the respondents were women (Table 1). Most of the study population (70.6%) resided in rural areas; 31.8% of older adults lived with spouses, children, and others; and 27.7% lived with children or children and others. Six percent of older adults lived alone and 5.4% of older adults lived with others only. More than half of the participants had less than a primary education. Twenty-two percent belonged to the poorest MPCE quintile and 16.5% of the respondents belonged to the richest MPCE quintile. Older adults reporting 1+ ADL limitations were 9.6%, while 29.2% of the respondents reported 1 chronic disease and 8.3% of respondents reported 3 or more chronic diseases. Overall, 7.9% of respondents had been hospitalized during the past year. The prevalence of depressive symptoms was 30.2% among the study population.

**Figure 1.**
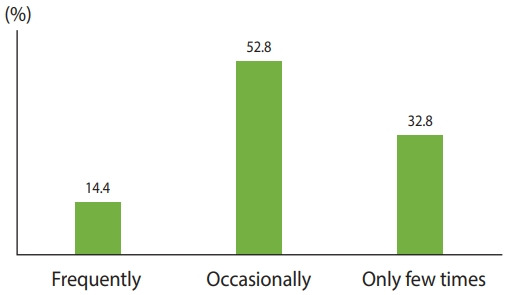
Regularity of abuse among older adults in India (n=1,269), Longitudinal Aging Study in India wave 1, 2017-2018.

Figure 1 shows the regularity of abuse among older adults in India. Overall, 14.4% had experienced abuse frequently, 52.8% occasionally, and the remaining 32.8% had experienced abuse only a few times.

**Figure 2.**
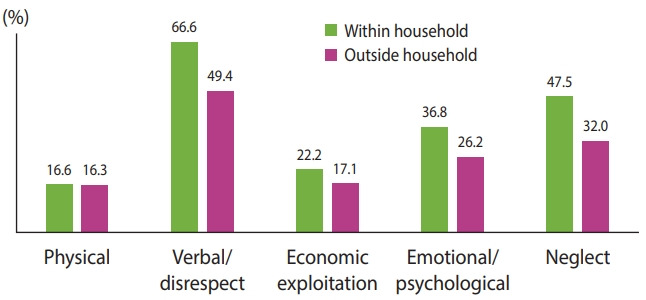
Type of abuse among older adults in India (n=1,002), Longitudinal Aging Study in India wave 1, 2017-2018.

Figure 2 shows types of elder abuse in India within and outside the household. Among those who reported elder abuse, the prevalence of verbal abuse or disrespect of older adults was 66.6% within the household and 49.4% outside of the household. The prevalence of neglect was 47.5% within the household and 32.0% outside of the household. Emotional/psychological abuse within the household was 36.8% and 26.2% outside of the household. Table 2 shows the prevalence of elder abuse by background characteristics. The overall prevalence of elder abuse was 5.2%. Approximately 3% of older adults had experienced abuse within the household. This study observed a higher prevalence (overall and within the household) of abuse among women. The prevalence of abuse was lower among currently married older adults. The prevalence of elder abuse was higher in rural areas. Older adults who lived alone reported a higher prevalence of abuse overall and within the household. A better socioeconomic status was associated with a lower prevalence of abuse among older adults. The results also showed that the prevalence of abuse was higher among older adults with ADL limitations and multimorbidity. The prevalence of abuse (overall) was slightly higher among those who had been hospitalized in the past year. The prevalence of depressive symptoms was higher among those who experienced abuse.

The prevalence of elder abuse overall was observed to be highest in Bihar (11.60%), as well as the prevalence of elder abuse within the household (6.79%) (Table 3). In 4 states of India, the prevalence of abuse was higher than the national average of 5.70%.

Older women experienced a higher likelihood of abuse, particularly within the household (Table 4). Older adults living alone were 2.09 times more likely to experience abuse (95% confidence interval [CI], 1.27 to 3.42; p< 0.001) compared with those living with spouses, children, and others. Similarly, older adults living with spouses and others were 41% more likely to experience abuse (95% CI, 1.20 to 1.65; p< 0.001) compared with those living with spouses, children and others. Older adults with less than a primary education are 83% more likely to experience abuse (95% CI, 1.20 to 2.77; p< 0.001) compared to those with a high school diploma or above. Similarly, elder abuse within the household was higher among those with lower educational levels. Older adults in the lowest MPCE quintile were 39% more likely to experience abuse (95% CI, 1.13 to 1.71; p< 0.001) than those in the highest MPCE quintile. Older adults with 1+ ADL limitations were 58% more likely to experience abuse (95% CI, 1.32 to 1.88; p< 0.001) and those with 3+ ADL limitations were 96% more likely to experience abuse (95% CI, 1.61 to 2.38; p< 0.001). Older adults with 1, 2 and 3+ chronic diseases were 31% (95% CI, 1.14 to 1.51; p<0.001), 35% (95% CI, 1.13 to 1.61; p< 0.001) and 43% (95% CI, 1.13 to 1.79; p< 0.001), respectively, more likely to experience abuse than those without any chronic disease. The association was even stronger for those who experienced abuse within a household. Our results showed a significant association between hospitalization and elder abuse. The gender-stratified regression results indicate a stronger association of socio-demographic and health factors with elder abuse among women. In particular, the association between socioeconomic status and elder abuse was high.

Older adults who experienced abuse were more likely to experience depressive symptoms, both overall and within the household (2.17 and 2.33 times, respectively) (Table 5).

## DISCUSSION

In this study, the overall prevalence of elder abuse was 5%, with a higher prevalence in the states of Bihar and Karnataka where more than 10% of older adults experienced abuse, suggesting a large regional heterogeneity in the prevalence of elder abuse. Gender differences in elder abuse were notable in that women experienced a higher likelihood of abuse. Education level showed a negative association with elder abuse, particularly among women. Health factors such as functional limitations and multimorbidity were significantly linked with elder abuse. The association between hospitalization and elder abuse was significant and positive. The association between depressive symptoms and elder abuse was very strong, suggesting the adverse effect of depression on elder abuse.

The overall prevalence of elder abuse was lower than shown in previous studies conducted in India [6,14,36] and other countries such as Singapore and Malaysia where the prevalence was 8.3% [13] and 9% [21], respectively, and the global prevalence was 17% (based on meta-analysis) [2]. The prevalence of elder abuse or ill-treatment was much higher in Taiwan [37] and China [38]. A study conducted in West Bengal, India showed a 26% prevalence of elder abuse [36]. Studies based on the nationally representative data of the Building a Knowledge Base on Population Aging in India survey showed a prevalence of 9% elder abuse. State-level variations indicate subnational differences in the prevalence of abuse [19,20] and may reflect underreporting of elder abuse in India. Most of the previous studies conducted in India, which showed higher prevalence rates used a validated scale to assess the prevalence of elder abuse [6,36]. In this respect, measuring the prevalence of elder abuse using validated scales is recommended.

State-level variations in the prevalence of elder abuse are important information for public policy. Creating awareness to prevent elder abuse and state-specific interventions targeting selected states will be useful, with a focus on older adults in low socioeconomic areas and those with chronic diseases or functional limitations.

In this study, gender differences in elder abuse were notable, with women experiencing a higher likelihood of elder abuse. These results are consistent with previous studies conducted in China [38], Korea [15], and the United States [16]. However, the results of this study contrast with previous studies conducted in India [14], which reported gender differences to be negligible or that more men experienced abuse. A study conducted in Malaysia also showed a higher likelihood of elder abuse among men [21]. Several studies that have studied men and women vulnerability in old age in India found a higher level of widowhood, lower educational attainment, poor living arrangements, and dependency among women [28,31]. In India, widowhood is higher among women primarily due to the high age gap between married men and women. While a high education level is one of the protective factors of elder abuse [14], older women have lower education levels than men. The large gap in education levels between women and men may be a reason for the higher levels of elder abuse among women. Most elderly women either depend on family members or live alone [39].

The association between education and elder abuse is significant, suggesting the protective role of education. The results of this study are consistent with those of previous studies on education and elder abuse conducted in India [14,19] and other countries [38,40]. Education as a human capital measure is one of the important factors in determining health, cognition, and well-being [41,42]. Previous studies conducted in India show that educated individuals perform well in cognition, functional independence, and handgrip strength [32,41]. These factors can reduce vulnerability and dependency, playing a significant role in lowering the prevalence of elder abuse. A higher level of education positively impacts economic independence, living environment, and level of openness, highlighting the role of education in preventing elder abuse.

In this study, health measurements showed a significant association with elder abuse. The results are consistent with previous studies in India where chronic diseases and functional limitations were closely linked to elder abuse [6,20,36]. Older adults with functional limitations face several adverse consequences including dependency on other family members for fulfilling their daily needs, which can increase the likelihood of elder abuse or ill-treatment [21]. There is a positive association between multimorbidity and elder abuse. Chronic conditions among older adults are associated with health-related problems and health expenditures [18,43,44] and older adults with multimorbidity are more likely to report functional limitations [43]. These factors increase the likelihood of dependency and a higher level of caregiving from other family members, as well as increasing health expenditures, contributing to an increased likelihood of elder abuse.

In this study, the odds of elder abuse were observed to be higher among those hospitalized within the past year, results consistent with existing studies [9]. However, the results can be interpreted as multidirectional, impacted by an increased level of caregiving after release from the hospital and other challenges associated with elder abuse, such as chronic disease and functional limitations. Overall, these factors can contribute to a higher likelihood of elder abuse. It is also possible that hospitalization increases financial distress in the household, which further increases the likelihood of elder abuse [45].

It is important to highlight health factors such as functional limitations. As a result of an aging population and demographic shifts, the prevalence of chronic diseases and functional limitations among the population is increasing [26]. Several studies have documented the adverse consequences of chronic diseases on subjective health and well-being [43]. The results of this study suggest that functional limitation plays a significant role in elder abuse with the disability-associated change in dependency an important driver of elder abuse.

In this study, a positive association between elder abuse and poor mental health is important to note. Older adults who experienced abuse were 2 times more likely to report depressive symptoms. This result is consistent with previous studies suggesting that elder abuse is closely linked with psychological distress [7] and depressive symptoms or depression [13,38]. In India, poor mental health conditions such as a high prevalence of depression and its associated consequences are strongly evident [46]. Elder abuse or neglect by family members has several effects on the overall well-being of older adults. Older adults in India have invested in their children and expect to be cared for in old age by their children or family members. However, when they experience elder abuse or neglect, feelings of rejection or loneliness can affect their mental health [10,47]. Reducing elder abuse has significant implications for improving the mental health of older adults.

This study had some limitations. The results of this study were based on cross-sectional data. The under-reporting of elder abuse is possible, affecting the overall prevalence of elder abuse. The presence of family members at the time of completing the survey could also affect the results of the study. Most studies conducted in developed countries used a validated elder abuse or ill-treatment scale, whereas this study did not use a scale to measure the prevalence of elder abuse. The use of a validated scale would be useful to understand the actual prevalence of elder abuse.

This is the first study to present the determinants of elder abuse by using the large-scale nationally representative LASI data on older adults. Furthermore, previous studies conducted in India on elder abuse have not focused on health and hospitalization variables. Health-related factors were included in this study, referencing literature that highlights the adverse consequences of poor health in old age.

## Figures and Tables

**Table 1.** Characteristics of the study population, Longitudinal Aging Study in India wave 1, 2017-2018

Characteristics	Weighted, %	Unweighted, n
Age (yr)		
60-69	58.5	18,974
70-79	30.2	9,101
≥80	11.3	3,389
Gender		
Men	47.5	15,098
Women	52.6	16,366
Marital status		
Currently married	61.6	19,658
Otherwise	38.4	11,317
Residence		
Rural	70.6	20,725
Urban	29.5	10,739
Living arrangement		
Living alone	5.7	1,609
Living with spouse/spouse and others	20.7	6,272
Living with children/children and others	27.7	8,258
Living with spouse and children only	8.7	3,140
Living with spouse, children and others	31.8	10,143
Living with others only	5.4	1,630
Education		
Less than primary	56.5	16,889
Primary completed	22.6	7,560
Higher secondary completed	16.8	5,560
Diploma or above	4.1	1,455
MPCE quintile		
Lowest /poorest	21.7	6,484
Second	21.7	6,477
Middle	21.0	6,416
Fourth	19.2	6,170
Highest/richest	16.5	5,917
ADL		
No	76.2	24,642
1	9.6	2,740
2	5.7	1,459
≥3	8.5	2,495
Multimorbidity (diseases)		
No	46.9	14,421
1	29.2	9,244
2	15.6	5,147
≥3	8.3	2,652
Hospitalisation		
No	92.1	28,940
Yes	7.9	2,518
Depressive symptoms		
No	69.8	21,911
Yes	30.2	8,506

MPCE, monthly per capita consumer expenditure; ADL, activities of daily living.

**Table 2.** Prevalence of elder abuse (weighted) by background characteristics, Longitudinal Aging Study in India wave 1, 2017-2018

Characteristics	Abuse (overall)	Abuse (within household)
Age (yr)		
60-69	5.1	2.7
70-79	5.8	3.4
≥80	4.2	2.8
Gender		
Men	4.8	2.3
Women	5.6	3.5
Marital status		
Currently married	4.9	2.5
Others	5.8	3.6
Residence		
Rural	5.8	3.1
Urban	3.8	2.5
Living arrangement		
Living alone	8.1	3.7
Living with others only	5.3	2.2
Living with spouse and others	5.9	3.1
Living with children/children and others	5.4	3.7
Living with spouse children and others	4.4	2.2
Education		
Less than primary	5.9	3.4
Primary completed	4.8	2.7
Higher secondary completed	4.1	2.0
Diploma or above	2.7	1.1
MPCE quintile		
Lowest /poorest	6.0	3.0
Second	5.4	2.9
Middle	5.7	3.5
Fourth	4.2	2.2
Highest/richest	4.6	2.8
ADL		
No	4.7	2.5
1	6.9	4.1
2	7.0	4.9
≥3	7.0	4.4
Multimorbidity (diseases)		
No	5.0	2.8
1	5.6	3.0
2	4.9	2.8
≥3	5.6	3.3
Hospitalisation		
No	5.2	2.9
Yes	5.6	2.9
Depressive symptoms		
No	3.7	2.1
Yes	8.7	4.9
Total	5.2	2.9

MPCE, monthly per capita consumer expenditure; ADL, activities of daily living.

**Table 3.** Prevalence of elder abuse (weighted) across states/UTs of India, Longitudinal Aging Study in India wave 1, 2017-2018

States/UTs	Abuse (overall)	Abuse (within household)
Jammu & Kashmir	2.07 (1.01, 3.12)	0.98 (0.23, 1.72)
Himachal Pradesh	1.09 (0.26, 1.92)	0.37 (-0.11, 0.86)
Punjab	2.09 (1.19, 2.99)	1.27 (0.56, 1.98)
Chandigarh	5.70 (3.29, 8.03)	3.80 (1.83, 5.78)
Uttarakhand	2.59 (1.35, 3.84)	0.84 (0.12, 1.56)
Haryana	3.40 (2.16, 4.64)	2.14 (1.15, 3.14)
Delhi	3.43 (1.81, 5.05)	2.86 (1.37, 4.35)
Rajasthan	3.24 (2.17, 4.31)	1.80 (0.99, 2.60)
Uttar Pradesh	6.43 (5.37, 7.49)	2.09 (1.46, 2.72)
Bihar	11.60 (10.10, 13.10)	6.79 (5.58, 8.00)
Arunachal Pradesh	4.09 (1.89, 6.29)	2.22 (0.56, 3.88)
Nagaland	0.26 (-0.15, 0.67)	0.26 (-0.15, 0.67)
Manipur	2.18 (0.99, 3.38)	1.13 (0.26, 2.00)
Mizoram	0.13 (-0.19, 0.46)	0.13 (-0.19, 0.46)
Tripura	1.64 (0.46, 2.82)	0.09 (-0.19, 0.38)
Meghalaya	0.77 (-0.08, 1.63)	0.77 (-0.07, 1.63)
Assam	2.91 (1.74, 4.08)	1.61 (0.73, 2.50)
West Bengal	7.55 (6.21, 8.90)	4.51 (3.43, 5.58)
Jharkhand	5.46 (4.14, 6.78)	2.70 (1.74, 3.65)
Odisha	2.85 (1.91, 3.79)	1.37 (0.70, 2.03)
Chhattisgarh	5.53 (3.90, 7.16)	1.97 (0.96, 2.98)
Madhya Pradesh	5.13 (3.91, 6.35)	3.67 (2.62, 4.72)
Gujarat	2.98 (1.89, 4.07)	2.47 (1.47, 3.46)
Daman & Diu	3.16 (1.47, 4.85)	2.09 (0.70, 3.48)
Dadra & Nagar Haveli	2.83 (1.26, 4.40)	1.95 (0.63, 3.26)
Maharashtra	3.93 (3.02, 4.85)	2.40 (1.67, 3.12)
Andhra Pradesh	2.06 (1.21, 2.92)	0.49 (0.07, 0.91)
Karnataka	10.10 (8.18, 12.00)	6.42 (4.83, 8.02)
Goa	1.28 (0.38, 2.17)	0.74 (0.05, 1.42)
Lakshadweep	-	-
Kerala	3.82 (2.71, 4.92)	1.93 (1.13, 2.73)
Tamil Nadu	2.37 (1.60, 3.15)	1.47 (0.86, 2.09)
Puducherry	1.64 (0.64, 2.65)	0.86 (0.12, 1.59)
Andaman & Nicobar Islands	1.27 (0.27, 2.26)	0.36 (-0.17, 0.90)
Telangana	2.09 (1.21, 2.97)	0.88 (0.30, 1.46)

Values are presented as % (95% confidence interval). UT, union territories.

**Table 4.** Multivariate logistic regression results^1^ of elder abuse in India, Longitudinal Aging Study in India wave 1, 2017-2018

Characteristics		Abuse	Abuse	Abuse (overall)	
				Men	Women
Age (yr)					
	60-69	1.00 (reference)	1.00 (reference)	1.00 (reference)	1.00 (reference)
	70-79	1.03 (0.90, 1.18)	1.12 (0.94, 1.35)	1.16 (0.95, 1.42)	0.97 (0.81, 1.16)
	≥80	0.79 (0.64, 0.99)^**^	0.95 (0.71, 1.26)	0.83 (0.59, 1.15)	0.79 (0.59, 1.05)
Gender					
	Men	1.00 (reference)	1.00 (reference)	-	-
	Women	1.37 (1.18, 1.59)^***^	1.83 (1.49, 2.25)^***^		
Marital status					
	Currently married	1.00 (reference)	1.00 (reference)	1.00 (reference)	1.00 (reference)
	Others	1.07 (0.67, 1.69)	1.57 (0.81, 3.05)	1.49 (0.76, 2.93)	0.86 (0.46, 1.60)
Residence					
	Rural	1.00 (reference)	1.00 (reference)	1.00 (reference)	1.00 (reference)
	Urban	0.70 (0.60, 0.82)^***^	0.72 (0.58, 0.89)^***^	0.72 (0.56, 0.91)^***^	0.73 (0.59, 0.89)^***^
Living arrangement					
	Living alone	2.09 (1.27, 3.42)^***^	1.06 (0.52, 2.18)	1.46 (0.67, 3.17)	2.66 (1.37, 5.19)^***^
	Living with others only	1.24 (0.74, 2.07)	0.66 (0.31, 1.39)	0.89 (0.40, 1.97)	1.55 (0.78, 3.06)
	Living with spouse and others	1.41 (1.20, 1.65)^***^	1.44 (1.16, 1.80)^***^	1.24 (0.99, 1.54)^*^	1.61 (1.27, 2.04)^***^
	Living with children/children and others	1.06 (0.67, 1.70)	0.78 (0.40, 1.53)	0.80 (0.40, 1.60)	1.32 (0.70, 2.50)
	Living with spouse children and others	1.00 (reference)	1.00 (reference)	1.00 (reference)	1.00 (reference)
Education					
	Less than primary	1.83 (1.20, 2.77)^***^	1.95 (1.10, 3.47)^**^	1.50 (0.93, 2.41)^*^	3.64 (1.31, 10.0)^**^
	Primary completed	1.87 (1.23, 2.84)^***^	1.86 (1.05, 3.32)^**^	1.73 (1.08, 2.76)^**^	3.40 (1.22, 9.48)^**^
	Higher secondary completed	1.50 (0.98, 2.29)^*^	1.53 (0.85, 2.75)	1.41 (0.88, 2.25)	2.38 (0.83, 6.81)
	Diploma or above	1.00 (reference)	1.00 (reference)	1.00 (reference)	1.00 (reference)
MPCE quintile					
	Lowest /poorest	1.39 (1.13, 1.71)^***^	1.26 (0.95, 1.67)	1.07 (0.79, 1.46)	1.66 (1.26, 2.19)^***^
	Second	1.27 (1.03, 1.55)^**^	1.24 (0.94, 1.63)	0.98 (0.72, 1.33)	1.52 (1.15, 2.00)^***^
	Middle	1.17 (0.96, 1.43)	1.27 (0.97, 1.65)^*^	1.17 (0.87, 1.56)	1.15 (0.87, 1.52)
	Fourth	1.06 (0.87, 1.31)	1.03 (0.78, 1.36)	0.79 (0.58, 1.08)	1.32 (1.00, 1.74)^**^
	Highest/richest	1.00 (reference)	1.00 (reference)	1.00 (reference)	1.00 (reference)
Work status					
	Currently working	1.00 (reference)	1.00 (reference)	1.00 (reference)	1.00 (reference)
	Worked in the past but currently not working	0.84 (0.73, 0.97)^**^	0.90 (0.74, 1.10)	0.85 (0.69, 1.04)	0.81 (0.66, 1.00)^*^
	Never worked	0.57 (0.47, 0.68)^***^	0.51 (0.39, 0.66)^***^	1.11 (0.71, 1.74)	0.50 (0.40, 0.63)^***^
ADL					
	No	1.00 (reference)	1.00 (reference)	1.00 (reference)	1.00 (reference)
	1	1.58 (1.32, 1.88)^***^	1.61 (1.27, 2.04)^***^	1.74 (1.32, 2.30)^***^	1.45 (1.15, 1.84)^***^
	2	1.37 (1.07, 1.75)^**^	1.34 (0.96, 1.86)^*^	1.38(0.90, 2.11)	1.35 (1.00, 1.83)^**^
	≥3	1.96 (1.61, 2.38)^***^	2.11 (1.65, 2.71)^***^	2.51 (1.86, 3.39)^***^	1.69 (1.31, 2.19)^***^
Multimorbidity (diseases)					
	No	1.00 (reference)	1.00 (reference)	1.00 (reference)	1.00 (reference)
	1	1.31 (1.14, 1.51)^***^	1.25 (1.03, 1.51)^**^	1.37 (1.11, 1.70)^***^	1.27 (1.06, 1.53)^***^
	2	1.35 (1.13, 1.61)^***^	1.40 (1.10, 1.77)^***^	1.41 (1.07, 1.85)^**^	1.32 (1.04, 1.67)^**^
	≥3	1.43 (1.13, 1.79)^***^	1.56 (1.16, 2.10)^***^	1.36 (0.95, 1.95)^*^	1.44 (1.06, 1.94)^**^
Hospitalisation					
	No	1.00 (reference)	1.00 (reference)	1.00 (reference)	1.00 (reference)
	Yes	1.31 (1.08, 1.60)^***^	1.29 (0.99, 1.68)^*^	1.11 (0.81, 1.51)	1.52 (1.17, 1.97)^***^
R^2^		0.081	0.087	0.082	0.088
Total		29,437	28,837	13,887	15,304

Values are presented as odds ratio (95% confidence interval). MPCE, monthly per capita consumer expenditure; ADL, activities of daily living.

1 Adjusted for states/union territories.

* p<0.05,

** p<0.01,

*** p<0.001.

**Table 5.** Multivariate logistic regression between elder abuse and depressive symptoms, Longitudinal Aging Study in India wave 1, 2017-2018

Abuse	OR (95% CI)^1^
Overrall	
No	1.00 (reference)
Yes	2.17 (1.92, 2.45)^***^
Within household	
No	1.00 (reference)
Yes	2.33 (1.97, 2.75)^***^

OR, odds ratio; CI, confidence interval.

1 Separate regression was used for each indicator; Results are adjusted for socio-demographic characteristics, health factors and states/union territories.

*** p<0.001.
